# Supplementary material for: Coordinated early immune response in the lungs is required for effective control of SARS-CoV-2 replication
Source: Nat Commun. 2025 Jun 25;16:5390. doi: 10.1038/s41467-025-60885-0 (PMC12198374; doi:10.1038/s41467-025-60885-0)
Supplement: Supplementary file 1 — Supplementary Information [file 41467_2025_60885_MOESM1_ESM.pdf]

Supplementary materials for

Coordinated early immune response in the lungs is required for effective control of SARS-CoV-2 replication

Authors

Klara Lenart, Hendrik Feuerstein, Mariana Marmorato, Laura Perez Vidakovics, Gerald McInerney, Mimi Guebre-Xabier, Jessica F. Trost, Bengt Eriksson, Gale Smith, Nita Patel, Karin Loré

Supplementary Figure 1

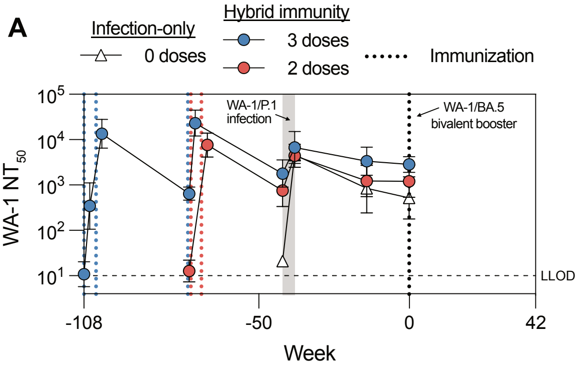

Supplementary Figure 1: Generation of hybrid immune rhesus macaques. Related to Fig 1.
(A) Longitudinal measurement of antibody responses throughout the preceding study until study start at week 106. NHPs were immunized at weeks 0, 4 and 35 (3 doses, blue group) or at weeks 35 and 39 (2 doses, red group). All animals were infected with SARS-CoV-2 WA-1 or P.1 at week 66 (n=3-6 biological replicates per group).
Data is presented as geometric mean ± geometric SD (A). Horizontal dotted line represents a lower level of detection (A). LLOD = lower level of detection. Source data are provided as a Source Data file.

Supplementary Figure 2

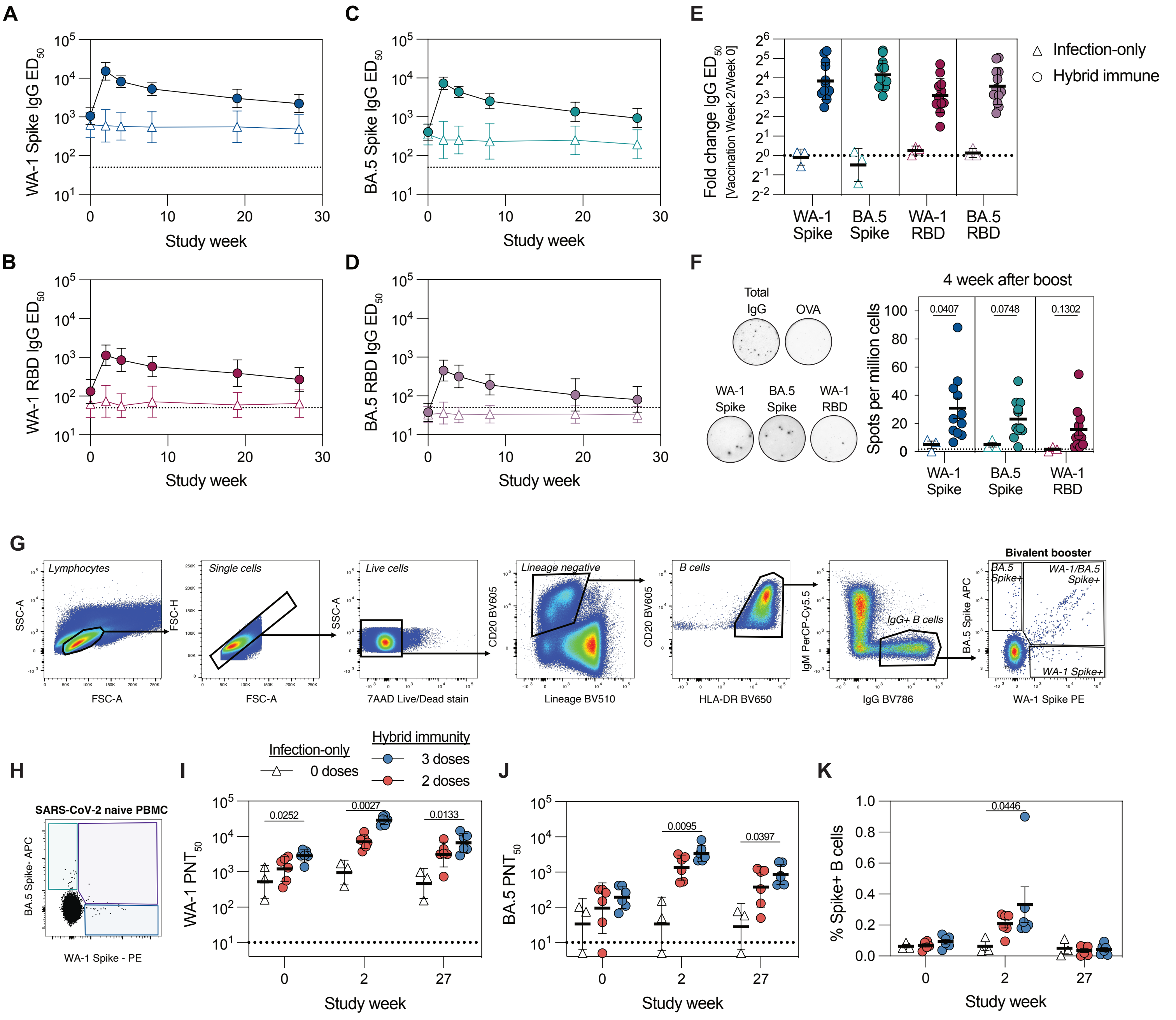

Supplementary Figure 2: B cell responses after bivalent booster. Related to Fig 1.
(A-D) IgG antibody binding titers against SARS-CoV-2 WA-1 Spike (A), WA-1 RBD (B), BA.5 Spike (C) and BA.5 RBD (D) after bivalent booster immunization at week 0 (n=3-12). Infected-only group was not immunized.
(E) Fold increase in IgG antibody binding titers two weeks after immunization compared to pre-immunization titers (n=3-12 biological replicates per group).
(F) Enumeration of WA-1 Spike, BA.5 Spike and WA-1 RBD-specific bone marrow plasma cells (BMPC). Representative ELISpot wells are shown on the left. All data is background subtracted based on OVA wells (n=3-12 biological replicates per group).
(G) Representative gating strategy for quantification of antigen-specific B cell responses.
(H) Representative flow cytometry plot depicting binding of IgG memory B cells to Spike probes in a naive NHP. Relates to data presented in Fig. 1G-I, Supplementary Fig. 1K and Fig. 3E-G.
(I-K) WA-1 (I) and BA.4/5 (J) serum neutralizing antibodies and Spike-binding memory B cell responses (K) to the bivalent booster based on the history of exposures to SARS-CoV-2 Spike (n=3-6 biological replicates per group).
Data is presented as geometric mean ± geometric SD (A-E, I, J) or arithmetic mean ± SEM (F, K). Horizontal dotted line represents a lower level of detection (A-D, F, I, J) or a fold change of 1 (E). Statistical analysis was performed using a non-parametric Kruskal-Wallis test with Dunn's correction for multiple analysis (F, I-K). **p<0.01, *p<0.05. Source data are provided as a Source Data file.

Supplementary Figure 3

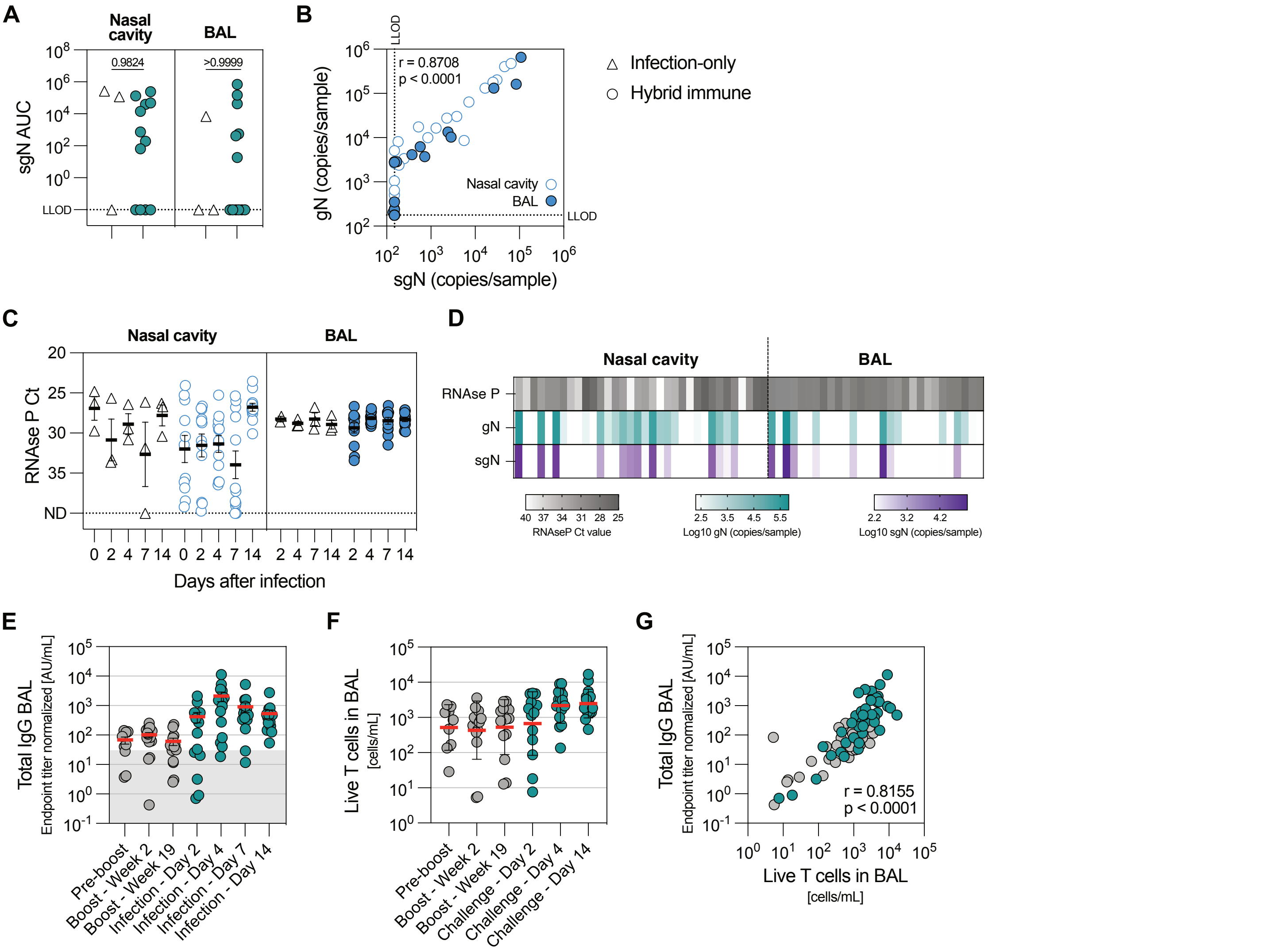

Supplementary Figure 3: Assessing the extent of SARS-CoV-2 infection in the respiratory tract. Related to Fig 2.

(A) Viral load as area under sgN curves (Fig. 2A) during the first 14 days after XBB.1.5 infection in the upper and lower airways (n=3–12 biological replicates per group).

(B) Correlation between the number of sgN and gN transcripts in upper and lower airways collected two and four days after infection.

(C) Detection of RNAseP mRNA transcripts in the nasal cavity and lungs (samples collected by nasal swabs and BAL, respectively) (n=3-12 biological replicates per group). RNAseP is an abundantly expressed ribozyme, the presence of which serves as quality control for sample collection and RNA extraction. ND = not detectable.

(D) Heatmap summarizing the number of RNAseP, gN and sgN transcripts in the nasal cavity and lungs samples collected at days 0, 2 and 4 after infection.

(E) Concentration of total IgG antibodies in the BAL fluid before and after XBB.1.5 infection (grey and green datapoints, respectively) (n=9-15 biological replicates per timepoint). Endpoint titers were normalized to the volume of retrieved BAL fluid. Grey shaded area denotes the samples with poor yield (total IgG endpoint titer < 1000), which were excluded from further analyses due to low accuracy.

(F) Concentration of T cells in the BAL fluid before and after XBB.1.5 infection (grey and green datapoints, respectively) (n=9-15 biological replicates per timepoint). Cell counts, collected from flow cytometry data, were normalized to the retrieved volume of BAL fluid.

(G) Correlation between concentration of T cells and IgG antibodies in the BAL fluid at all tested timepoints. Data is presented as arithmetic mean ± SEM (C) or geometric mean ± geometric SD (E, F). Horizontal dotted line represents a lower level of detection (A-C). Vertical dotted line represents a lower level of detection (B). Statistical analysis was performed using a non-parametric Kruskal-Wallis test (A) or Spearman correlation (B, G). LLOD = lower level of detection. AUC = area under curve. Source data are provided as a Source Data file.

Supplementary Figure 4

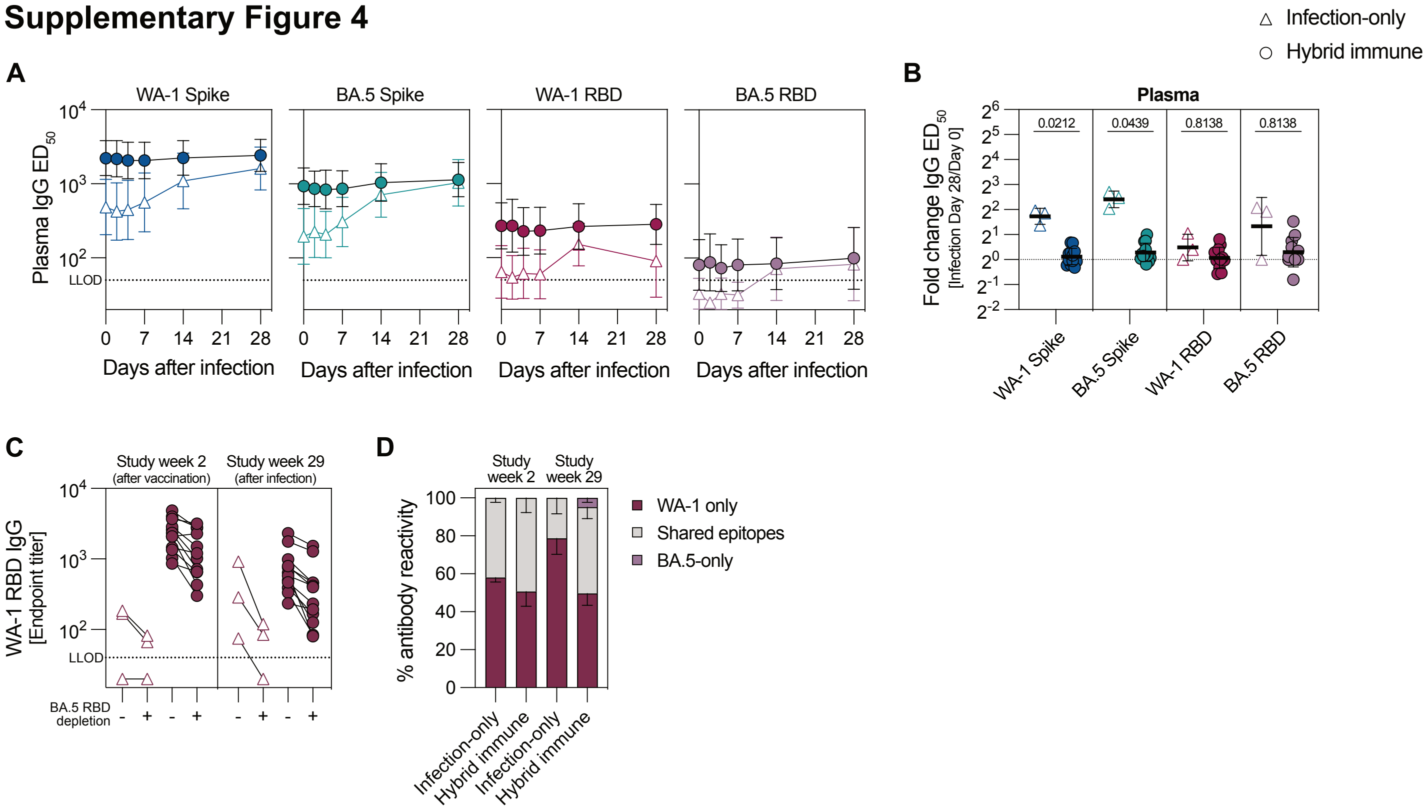

Supplementary Figure 4: Peripheral B cell responses to XBB.1.5 infection. Related to Fig 3.
(A) IgG antibody binding titers against SARS-CoV-2 WA-1 Spike, WA-1 RBD, BA.5 Spike and BA.5 RBD after XBB.1.5 infection (n=3-12 biological replicates per group).
(B) Fold increase in IgG antibody binding titers four weeks after infection compared to pre-infection titers (n=3-12 biological replicates per group).
(C) Binding of plasma IgG antibodies to WA-1 RBD with and without depletion of BA.5 RBD-specific antibodies, two weeks after bivalent booster and XBB.1.5 infection (n=3–12 biological replicates per group).
(D) Frequency of plasma IgG antibodies against RBD epitopes that are present either only on WA-1 RBD, only on BA.5 RBD or shared between both protein variants (n=3–12 biological replicates per group).
Data is presented as geometric mean ± geometric SD (A-B) and as arithmetic mean ± SEM (D). Horizontal dotted line represents a lower level of detection (A) or a fold change of 1 (B). Statistical analysis was performed using a non-parametric Spearman correlation (B). LLOD = lower level of detection. Source data are provided as a Source Data file.

Supplementary Figure 5

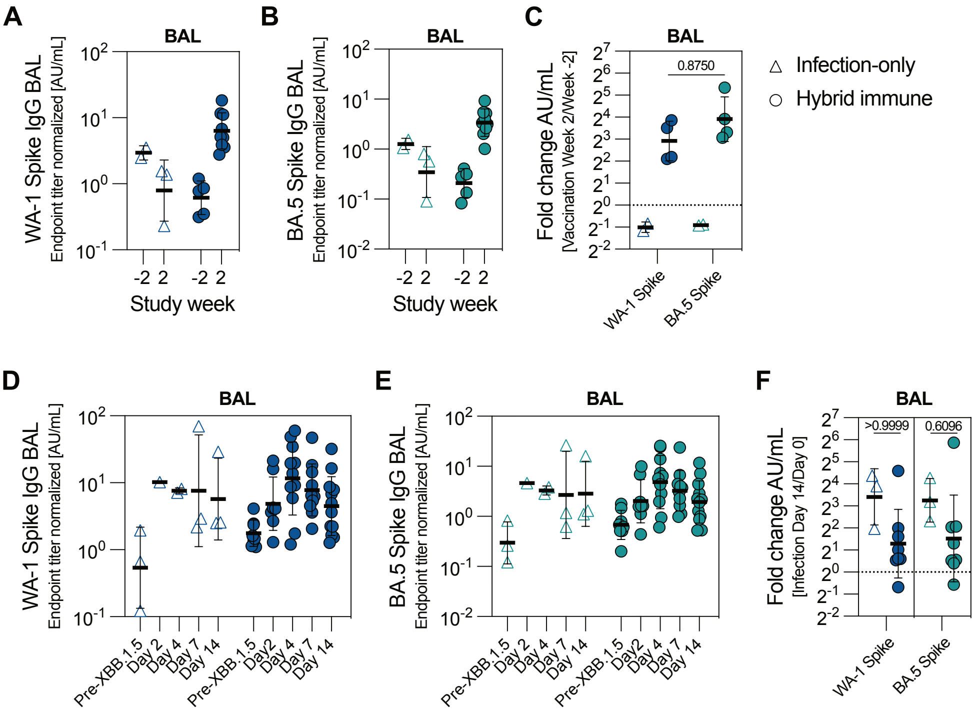

Supplementary Figure 5: Mucosal antibody responses after bivalent immunization and XBB.1.5 infection. Related to Fig 4.
(A, B) WA-1 S- (A) and BA.5 S-specific IgG antibodies (B) in the BAL fluid after immunization (n=2–12 biological replicates per group).
(C) Fold increase in S-specific IgG antibody titers in the BAL fluid two weeks after bivalent immunization compared to pre-immunization timepoint (n=2–4 biological replicates per group).
(D, E) WA-1 S- (D) and BA.5 S-specific IgG antibodies (E) in the BAL fluid after XBB.1.5 infection (n=2–12 biological replicates per group).
(F) Fold increase in S-specific IgG antibody titers in the BAL fluid two weeks after XBB.1.5 infection compared to before infection (n=3–8 biological replicates per group).
All endpoint and neutralizing titers in the BAL are normalized to the retrieved volume of the BAL fluid. Samples with total IgG endpoint titer < 1000 (Fig. S3D) were excluded from the analysis. Data is presented as geometric mean ± geometric SD (A-F). Horizontal dotted line represents a fold change of 1 (C, F). Statistical analysis was performed using a non-parametric Wilcoxon test (C) or a non-parametric Kruskal-Wallis test with Dunn’s correction for multiple analysis (F). Source data are provided as a Source Data file.

Supplementary Figure 6

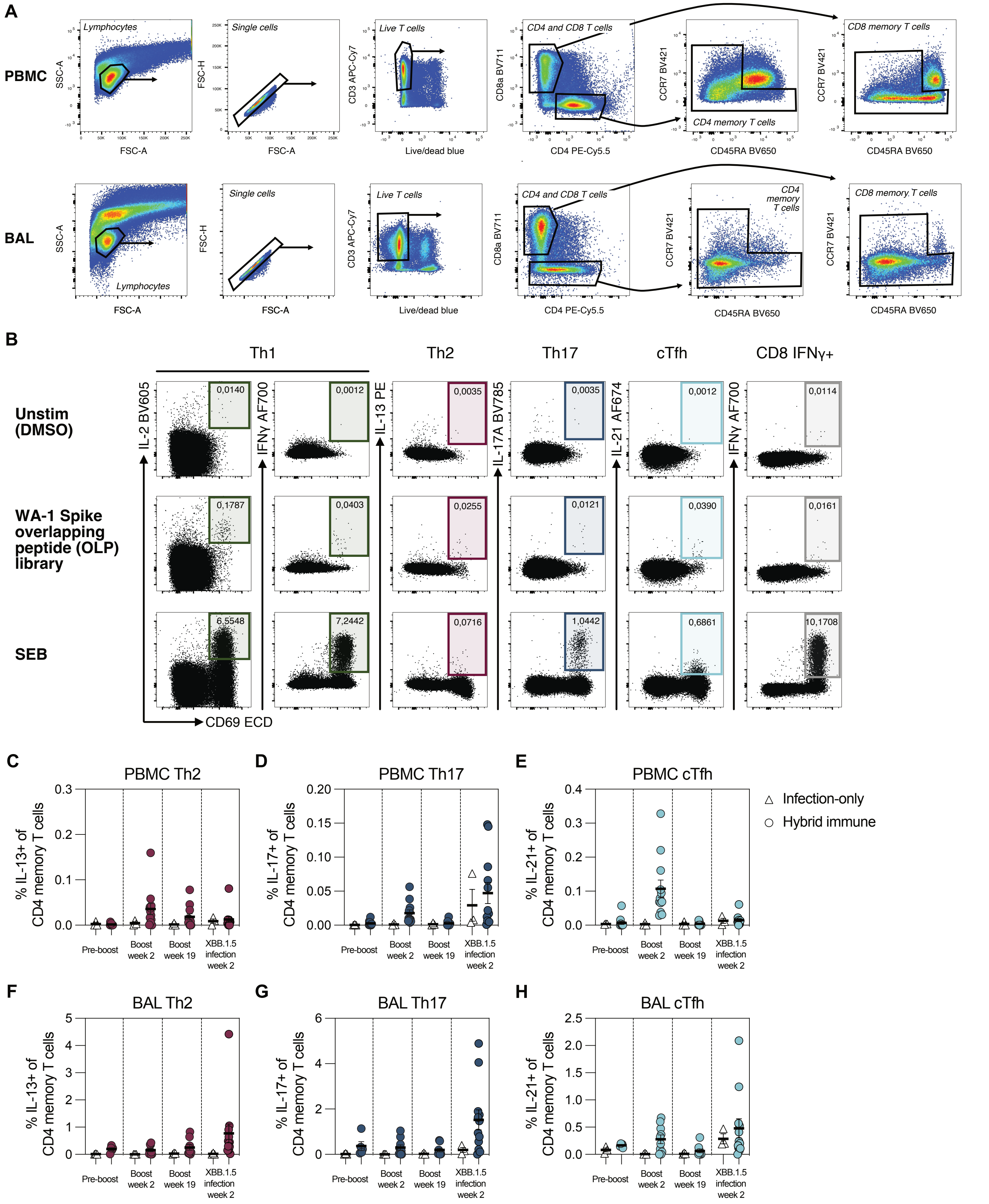

Supplementary Figure 6: T cell responses after bivalent boost and XBB.1.5 infection. Related to Fig 5.
(A-B) Representative gating strategies used to define CD4 and CD8 memory T cells in blood and BAL (A), and quantify Spike-specific memory T cell subsets in the respective tissues (B). Background subtraction was performed based on DMSO condition. Staphylococcal enterotoxin B (SEB) served as positive control. Relates to data presented in Fig. 5, Fig. 7A-K and Supplementary Fig. 8.
(C-E) Spike-specific Th2 (C), Th17 (D) and cTfh (E) memory T cells in the blood before and after bivalent immunization and XBB.1.5 infection (n=3-12 biological replicates per group).
(F-H) Spike-specific Th2 (F), Th17 (G) and cTfh (H) memory T cells in the BAL before and after bivalent immunization and XBB.1.5 infection (n=3-12 biological replicates per group).
Data is presented as arithmetic mean ± SEM (C-H). Source data are provided as a Source Data file.

Supplementary Figure 7

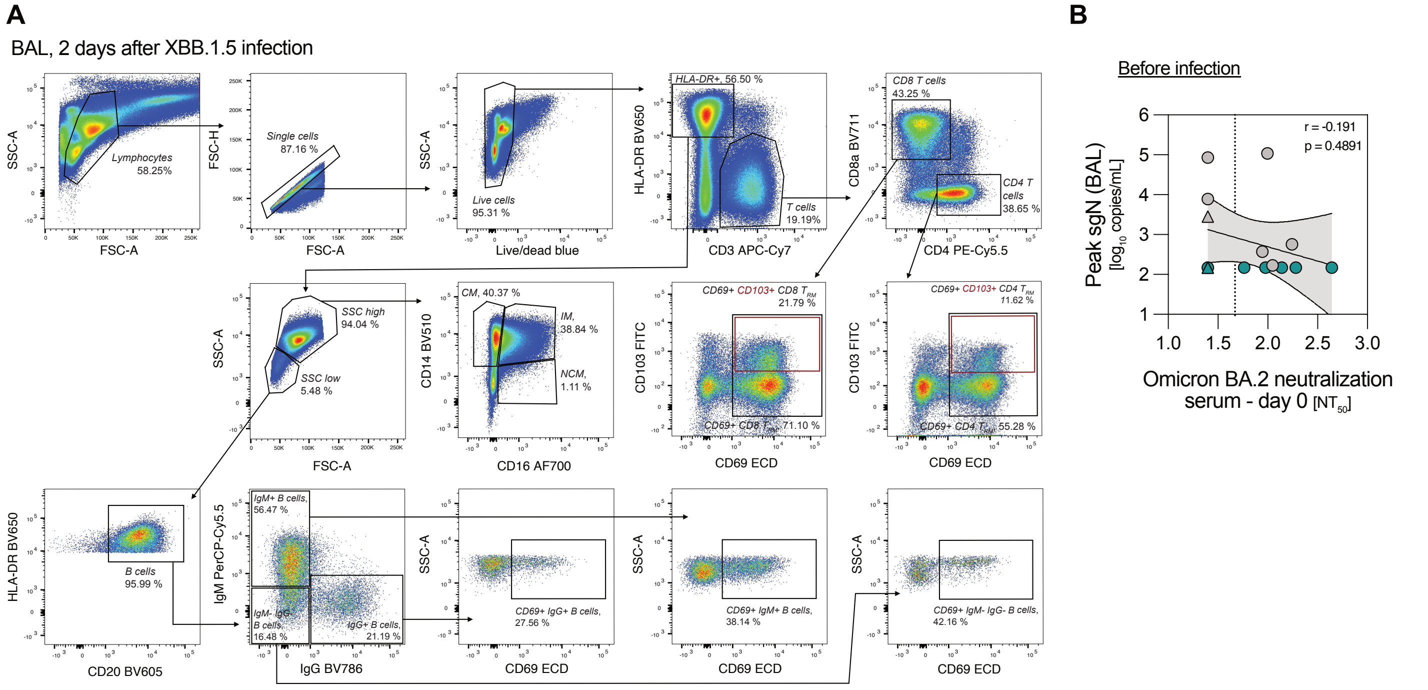

Supplementary Figure 7: Expansion of S-specific lung tissue-resident CD8 memory T cells is associated with protection from infection. Related to Fig 6.
(A) Representative gating strategy to identify different immune cell subsets in the BAL after infection. Related to data presented in Fig. 6D-M.
(B) Correlation between Omicron BA.2 neutralization in the blood before infection and peak sgN viral loads in BAL after infection (n=15).
Statistical analysis was performed using a non-parametric Spearman correlation (B). Source data are provided as a Source Data file.

Supplementary Figure 8

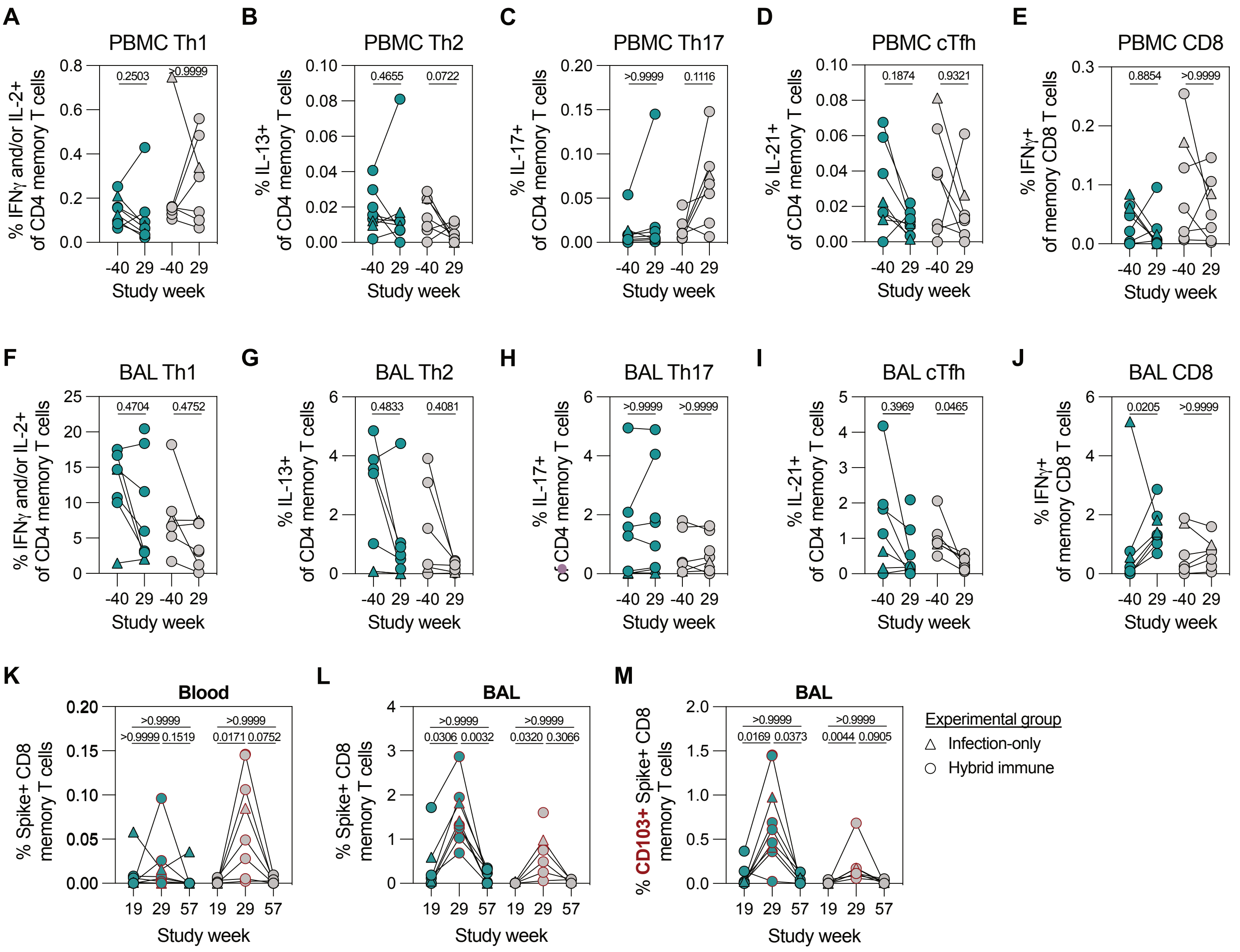

Supplementary Figure 8: T cell responses after WA-1/P.1 infection and XBB.1.5 re-infection. Related to Fig 7.

(A-E) Spike-specific Th1 (A), Th2 (B), Th17 (C), cTfh (D) and CD8 (E) memory T cells in the blood after WA-1/P.1 and XBB.1.5 infection (n=7-8 biological replicates per group).

(F-J) Spike-specific Th1 (F), Th2 (G), Th17 (H), cTfh (I) and CD8 (J) memory T cells in the BAL after WA-1/P.1 and XBB.1.5 infection (n=7-8 biological replicates per group).

(K-M) Spike-specific CD8 T cells responses in blood (K) and BAL (L, M) before the XBB.1.5 infection, two weeks and 30 weeks after (weeks 19, 29 and 57, respectively) (n=7-8 biological replicates per group). Statistical analysis was performed using a non-parametric Kruskal-Wallis test with Dunn's correction for multiple analysis (A-M). *$p<0.05$, ns = not significant. Source data are provided as a Source Data file.

Supplementary Figure 9

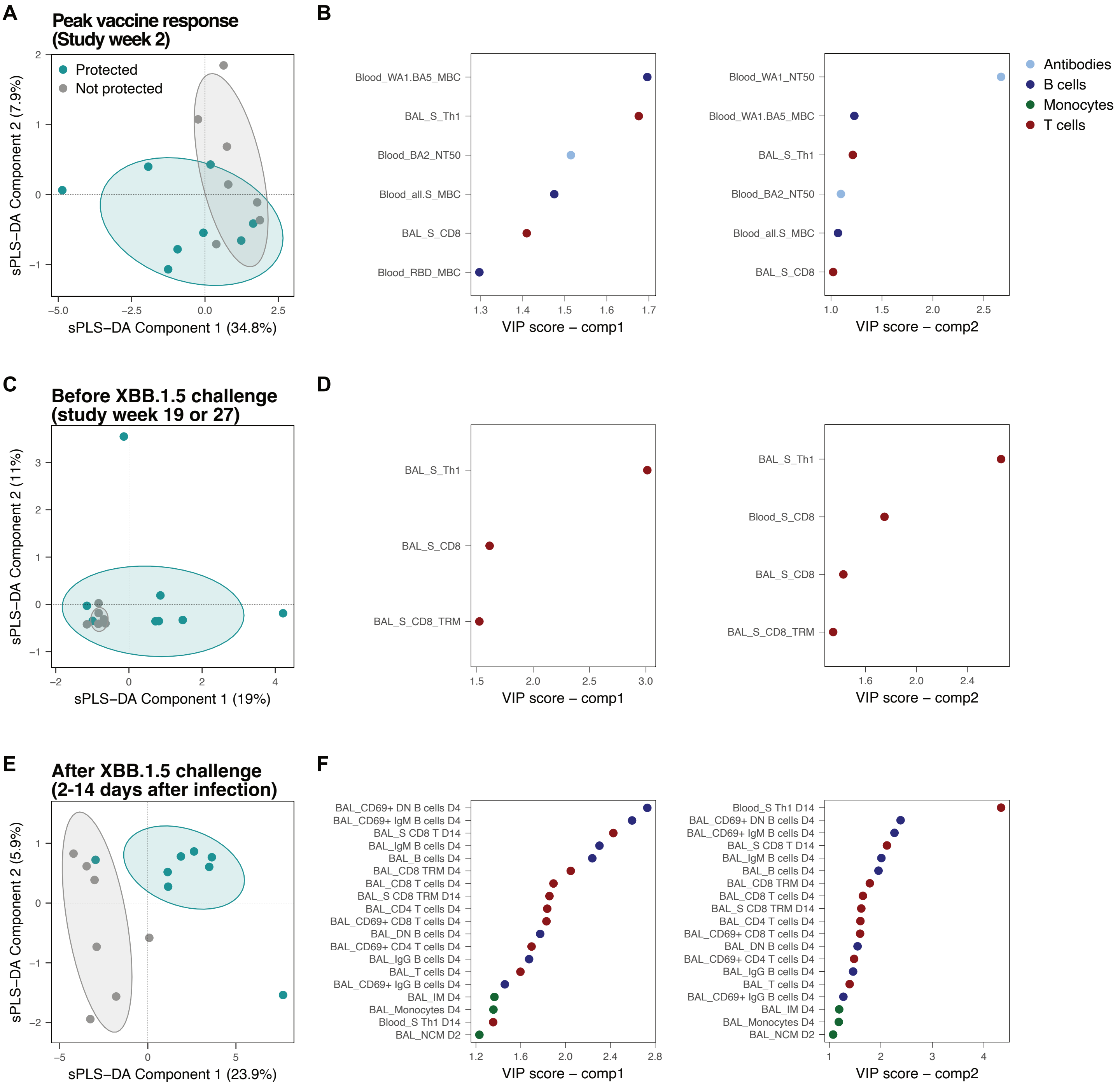

Supplementary Figure 9: sPLS-DA analysis at different timepoints. Related to Fig 7.
(A-F) sPLS-DA analysis and contribution of different immune parameters measured in blood and BAL to sPLS-DA components 1 and 2 at different study timepoints: peak vaccine immunity (including 15 different variables, A-B), before XBB.1.5 infection (14 different variables, C-D) and after XBB.1.5 infection (73 different variables, E-F). MBC = memory B cell, IM = intermediate monocyte, NCM = non-classical monocyte.

Supplementary Table 1: Detailed description of SARS-CoV-2 exposure history for each NHP included in the study.

Study group	NHP ID	Study week						
		-108	-104	-73	-69	-42 (infection)	0	27 (infection)
Hybrid immune	J01	WA-1 S	WA-1 S	P.1 S		WA-1 inf	WA-1/BA.5 S	XBB.1.5 inf
	J02	WA-1 S	WA-1 S	P.1 S		P.1 inf	WA-1/BA.5 S	XBB.1.5 inf
	J08	WA-1 S	WA-1 S	P.1 S		P.1 inf	WA-1/BA.5 S	XBB.1.5 inf
	J11	WA-1 S	WA-1 S	P.1 S		P.1 inf	WA-1/BA.5 S	XBB.1.5 inf
	J16	WA-1 S	WA-1 S	P.1 S		WA-1 inf	WA-1/BA.5 S	XBB.1.5 inf
	J17	WA-1 S	WA-1 S	P.1 S		WA-1 inf	WA-1/BA.5 S	XBB.1.5 inf
	J05			WA-1 S	WA-1 S	P.1 inf	WA-1/BA.5 S	XBB.1.5 inf
	J10			WA-1 S	WA-1 S	P.1 inf	WA-1/BA.5 S	XBB.1.5 inf
	J18			WA-1 S	WA-1 S	P.1 inf	WA-1/BA.5 S	XBB.1.5 inf
	J06			P.1 S	P.1 S	P.1 inf	WA-1/BA.5 S	XBB.1.5 inf
	J09			P.1 S	P.1 S	P.1 inf	WA-1/BA.5 S	XBB.1.5 inf
	J13			P.1 S	P.1 S	P.1 inf	WA-1/BA.5 S	XBB.1.5 inf
Infection-only	J07					P.1 inf		XBB.1.5 inf
	J12					P.1 inf		XBB.1.5 inf
	J15					WA-1 inf		XBB.1.5 inf

Legend:

WA-1 S = WA-1 Spike vaccination (NVX-CoV2373)
P.1 S = WA-1 Spike vaccination (NVX-CoV2443)
WA-1 inf = WA-1 SARS-CoV-2 infection
P.1 inf = P.1 SARS-CoV-2 infection
WA-1/BA.5 S = WA-1/ BA.5 bivalent Spike vaccination (50% NVX-CoV2373 and 50% NVX-COV2540)
XBB.1.5 inf = XBB.1.5 SARS-CoV-2 infection

Supplementary Table 2: Antibody cocktails for flow cytometry.

Memory B cell panel

Name	Clone	Vendor and catalogue number	Dilution
IgM PerCP-Cy5.5	G20-127	BD, #561285	1:40
CD3 BV510	SP34-2	BD, #740187	1:40
CD123 BV510	6H6	Biolegend, #306022	1:40
CD16 BV510	3G8	BD, #563830	1:80
HLA-DR BV650	L243	Biolegend, #307650	1:80
IgG BV786	G18-145	BD, #564230	1:80
CD20 BV605	2H7	Biolegend, #302334	1:160
CD14 BV510	M5E2	Biolegend, #301842	1:160
IgD FITC	Polyclonal	Southern Biotech, #2030-02	1:167

T cell re-stimulation panel

Surface staining

Name	Clone	Vendor and catalogue number	Dilution
CD103 FITC	2G5	Beckman Coulter, #B49222	1:50
CCR7 BV421	G043H7	Biolegend, #353208	1:50
CD8a BV711	RPA-T8	Biolegend, #301044	1:80
CD4 PE-Cy55	S3.5	Invitrogen, #MHCD0418	1:80
CD45RA BV650	5H9	BD, #740608	1:500

Intracellular staining

Name	Clone	Vendor and catalogue number	Dilution
IL-21 AF647	3A3-N2.1	BD, #560493	1:20
IL-13 PE	JES10-5A2	BD, #559328	1:33
IL-2 BV605	MQ1-17H12	BD, #564165	1:50
IL-17A BV785	BL168	Biolegend, #512338	1:67
CD69 ECD	TP1.55.3	Beckman Coulter, #6607110	1:67
CD3 APC-Cy7	SP34.2	BD, #557757	1:200
IFNg AF700	B27	Biolegend, #506516	1:200

Innate and adaptive panel for BAL staining

Name	Clone	Vendor and catalogue number	Dilution
IgM PerCP-Cy5.5	G20-127	BD, #561285	1:40
CD103 FITC	2G5	Beckman Coulter, #B49222	1:50
CD3 APC-Cy7	SP34.2	BD, #557757	1:50
CD69 ECD	TP1.55.3	Beckman Coulter, #6607110	1:67
HLA-DR BV650	L243	Biolegend, #307650	1:80
IgG BV786	G18-145	BD, #564230	1:80
CD8a BV711	RPA-T8	Biolegend, #301044	1:80
CD4 PE-Cy5	SK3	BD, #566925	1:100
CD14 BV510	M5E2	Biolegend, #301842	1:125
CD16 AF700	38G	BD, #560713	1:125
CD20 BV605	2H7	Biolegend, #302334	1:160

Supplementary Table 3: Primer and probe sequences for RT-qPCR.

Target	Name	Sequence (5' → 3')
gN	N_Sarbeco_F	CAC ATT GGC ACC CGC AAT C
	N2_P	5'FAM-CGA TCA AAA CAA CGT CGG CCC C-3'BHQ1
	wtN_R	GGT GAA CCA AGA CGC AGT AT
sgN	sgLeadSARSCoV2_F	CGA TCT CTT GTA GAT CTG TTC TC
	N2_P	5'FAM-CGA TCA AAA CAA CGT CGG CCC C-3'BHQ1
	wtN_R	GGT GAA CCA AGA CGC AGT AT
RNAse P	RNAseP_rh_F	AGA CTT GGA CGT GCG AGC G
	RNAseP_rh_R	GAG CCG CTG TCT CCA CAA GT
	RNAseP_rh_P	5'FAM/ZEN-TTC TGA CCT GAA GGC TCT GCG CG-3'IBFQ
