# Supplementary material for: Coordinated early immune response in the lungs is required for effective control of SARS-CoV-2 replication
Source: Nat Commun. 2025 Jun 25;16:5390. doi: 10.1038/s41467-025-60885-0 (PMC12198374; doi:10.1038/s41467-025-60885-0)
Supplement: Supplementary file 2 — Reporting Summary [file 41467_2025_60885_MOESM2_ESM.pdf]

nature portfolio

Corresponding author(s): Karin Loré

Last updated by author(s): May 22, 2025

Reporting Summary

Nature Portfolio wishes to improve the reproducibility of the work that we publish. This form provides structure for consistency and transparency in reporting. For further information on Nature Portfolio policies, see our Editorial Policies and the Editorial Policy Checklist.

Statistics

For all statistical analyses, confirm that the following items are present in the figure legend, table legend, main text, or Methods section.

n/a	Confirmed	
☐	☒	The exact sample size (*n*) for each experimental group/condition, given as a discrete number and unit of measurement
☐	☒	A statement on whether measurements were taken from distinct samples or whether the same sample was measured repeatedly
☐	☒	The statistical test(s) used AND whether they are one- or two-sided<br>*Only common tests should be described solely by name; describe more complex techniques in the Methods section.*
☐	☒	A description of all covariates tested
☐	☒	A description of any assumptions or corrections, such as tests of normality and adjustment for multiple comparisons
☐	☒	A full description of the statistical parameters including central tendency (e.g. means) or other basic estimates (e.g. regression coefficient) AND variation (e.g. standard deviation) or associated estimates of uncertainty (e.g. confidence intervals)
☐	☒	For null hypothesis testing, the test statistic (e.g. *F*, *t*, *r*) with confidence intervals, effect sizes, degrees of freedom and *P* value noted<br>*Give P values as exact values whenever suitable.*
☒	☐	For Bayesian analysis, information on the choice of priors and Markov chain Monte Carlo settings
☒	☐	For hierarchical and complex designs, identification of the appropriate level for tests and full reporting of outcomes
☒	☐	Estimates of effect sizes (e.g. Cohen's *d*, Pearson's *r*), indicating how they were calculated

Our web collection on statistics for biologists contains articles on many of the points above.

Software and code

Policy information about availability of computer code

Data collection	Flow cytometry data was collected using commercially available BD FACSDiva software (v8.1 or 9.1, Beckton Dickinson). ELISA data was collected using commercially available SkanIt software (v 7.0, Thermo Fisher). Luminescence was measured using a SpectraMax iD3 microplate reader with SoftMax Pro GxP software (v6.5.1, Molecular Devices). ELISpot plates were read using the AID ELISpot reader (Autoimmun Diagnostika). RT-qPCR data was acquired using CFX384 Touch Real-Time PCR Detection System equipped with CFX Manager software (v3.1, BioRad).
Data analysis	Flow cytometry data analysis was performed using Flowjo (v 10.8.2).<br>Statistical analyses were performed using Graphpad Prism (v.10.0) or R (v.4.2.1) for Mac OS X.

For manuscripts utilizing custom algorithms or software that are central to the research but not yet described in published literature, software must be made available to editors and reviewers. We strongly encourage code deposition in a community repository (e.g. GitHub). See the Nature Portfolio guidelines for submitting code & software for further information.

Data

Policy information about availability of data

All manuscripts must include a data availability statement. This statement should provide the following information, where applicable:

- Accession codes, unique identifiers, or web links for publicly available datasets
- A description of any restrictions on data availability
- For clinical datasets or third party data, please ensure that the statement adheres to our policy

All data supporting the findings of this study are available within the paper and its Supplementary Information.

Research involving human participants, their data, or biological material

Policy information about studies with human participants or human data. See also policy information about sex, gender (identity/presentation), and sexual orientation and race, ethnicity and racism.

Reporting on sex and gender	N/A
Reporting on race, ethnicity, or other socially relevant groupings	N/A
Population characteristics	N/A
Recruitment	N/A
Ethics oversight	N/A

Note that full information on the approval of the study protocol must also be provided in the manuscript.

Field-specific reporting

Please select the one below that is the best fit for your research. If you are not sure, read the appropriate sections before making your selection.

☒ Life sciences ☐ Behavioural & social sciences ☐ Ecological, evolutionary & environmental sciences

For a reference copy of the document with all sections, see nature.com/documents/nr-reporting-summary-flat.pdf

Life sciences study design

All studies must disclose on these points even when the disclosure is negative.

Sample size	The number of non-human primates used was based on the number of animals available and standard practice in the field.
Data exclusions	Data points deemed unreliable due to technical experimental issues were excluded in few cases. Bronchoalveolar samples that contained less than 1000 live T cells or total IgG endpoint titer < 1000 were excluded due to suboptimal sampling.
Replication	Due to limitations in large animal trials, non-human primate immunization experiments were not replicated. All the samples were run as technical duplicates when possible, this includes all the ELISAs and the neutralization assays performed in this study. All attempts at replication were successful.
Randomization	Animals were not randomly assigned to the groups. The initial vaccination grouping was done controlling for sex and weight in order to have similar distribution of these factors between the study groups, and in this study the animals were assigned to groups based on experimental treatment in the preceding study.
Blinding	Investigators were not blinded to data collection and/or data analysis due to lack of personnel and resources.

Reporting for specific materials, systems and methods

We require information from authors about some types of materials, experimental systems and methods used in many studies. Here, indicate whether each material, system or method listed is relevant to your study. If you are not sure if a list item applies to your research, read the appropriate section before selecting a response.

Materials & experimental systems

n/a	Involved in the study
☐	☒ Antibodies
☐	☒ Eukaryotic cell lines
☒	☐ Palaeontology and archaeology
☐	☒ Animals and other organisms
☒	☐ Clinical data
☒	☐ Dual use research of concern
☒	☐ Plants

Methods

n/a	Involved in the study
☒	☐ ChIP-seq
☐	☒ Flow cytometry
☒	☐ MRI-based neuroimaging

Antibodies

Antibodies used	Information about all the antibodies used in the flow cytometry panels, ELISA and ELISpot assays are described in the Methods section or in the Supplementary tables.
Validation	All antibodies used in this study were commercial antibodies. Reactivity of all antibody reagents was based on manufacturer's information on manufacturer's website: https://www.bd.com/en-us; https://www.biolegend.com/en-us; https://www.thermofisher.com/us/en/home.html; https://www.miltenyibiotec.com/; https://www.beckmancoulter.com/; https://www.southernbiotech.com/; https://www.jacksonimmuno.com/.<br><br>Cross-reactivity with rhesus surface markers was checked against NHP Reagent Resource Reactivity Database operated by NIH (https://www.nhpreagents.org/ReactivityDatabase).<br><br>Flow antibodies were titrated by single staining rhesus PBMCs with a 7-step 2-fold dilution series starting at 1:20 and a no-antibody control, and selecting the antibody titer that showed best signal-to-noise ratio.

Eukaryotic cell lines

Policy information about cell lines and Sex and Gender in Research

Cell line source(s)	Vero E6 (ECACC), HEK293T/hACE2 (Creative Biogene).
Authentication	Vero and HEK cells are used routinely for SARS-CoV-2 related neutralization assays. Vero E6 cells were speciated by DNA barcoded sequencing of the mitochondria cytochrome c oxidase subunit 1 gene by ECACC. HEK293T/hACE2 cell line was not authenticated after purchase.
Mycoplasma contamination	Tested by PCR and Hoechst 33258 by ECACC; mycoplasma not detected for Vero E6. Mycoplasma not detected for HEK293T, tested by Creative Biogene.
Commonly misidentified lines (See ICLAC register)	None listed in ICLAC register for Vero E6 or HEK293T.

Animals and other research organisms

Policy information about studies involving animals; ARRIVE guidelines recommended for reporting animal research, and Sex and Gender in Research

Laboratory animals	Fifteen Indian rhesus macaques (Macaca mulatta) of approximate 4-6 years of age.
Wild animals	This study did not involve wild animals.
Reporting on sex	Fifteen Indian rhesus macaques (Macaca mulatta), seven females and eight males.
Field-collected samples	This study did not involve field-collected samples.
Ethics oversight	This study was approved by the Stockholm Regional Ethical Board on Animal Experiments (16344-2017, 18427-2019).

Note that full information on the approval of the study protocol must also be provided in the manuscript.

Flow Cytometry

Plots

Confirm that:

- [x] The axis labels state the marker and fluorochrome used (e.g. CD4-FITC).
- [x] The axis scales are clearly visible. Include numbers along axes only for bottom left plot of group (a 'group' is an analysis of identical markers).
- [x] All plots are contour plots with outliers or pseudocolor plots.
- [x] A numerical value for number of cells or percentage (with statistics) is provided.

Methodology

Sample preparation

PBMC were isolated using a Ficoll density gradient protocol and either cryopreserved or used directly. BAL cells were washed with PBS, filtered through 70um cell strainer before use and used fresh. PBMCs were stained with fluorescently labeled antibodies for analysis of innate immune cells and memory B cells. For T cell assays, PBMCs and BAL cells were stimulated with antigenic peptides as described in the methods section and then stained with a panel of fluorescent antibodies for analysis of intracellular cytokines.

Instrument

Samples were acquired on a BD LSRFortessa flow cytometer.

Software

Data was acquired with BD FACSDiva (Beckton Dickinson) version 8.0.1 or 9.0.1, and analyzed using Flowjo version 10 (BD Life Sciences).

Cell population abundance

NA

Gating strategy

Details regarding the gating strategy for each of the subset studied is described in detail in the supplementary material with representative figures.

Memory B cells were defined as live single CD3- CD11c- CD14- CD16- CD123- CD20+ HLA-DR+ IgM- IgG+ cells, out of which we defined Spike-specific memory B cells as cells that are double positive for the fluorescently labelled Spike probes in two colors (APC and PE). Within the Spike-specific population, the B cells that also bound fluorescently labelled RBD were defined as RBD-specific.

CD4/CD8+ memory T cells were first identified as live single CD3+ CD8- CD4+ NOT(CD45RA+CCR7+) cells for CD4 memory and live single CD3+ CD4- CD8+ NOT(CD45RA+CCR7+) cells for CD8 memory. Then, activated CD69+ cells were analyzed for the presence of different intracellular cytokines; IFNg, IL-2, IL-17A, IL-13 and IL-21. Lung tissue-resident memory cells were defined as non-naive (NOT(CD45RA+CCR7+)) CD3+ CD4+ or CD8+ T cells that express CD69+ and CD103+.

In the BAL phenotyping panel, T cells were defined as live single CD3+ HLA-DR- cells that express either CD4 (CD4 T cells) or CD8 (CD8 T cells). Both subsets were further assessed for the tissue-resident phenotype (CD69+CD103+) or just activated phenotype (CD69+). B cells were defined as live single HLA-DR+ CD3- cells, SSC-A low and CD20+. They were further assessed for Ig isotype (IgG+, IgM+ or double negative), and each isotype-subset was assessed for CD69 expression. Monocytes were defined as live single HLA-DR+ CD3- SSC-Ahigh cells. Classical monocytes are CD14+CD16-, intermediate monocytes are CD14 +, D16+ and non-classical monocytes are CD16+CD14-.

- [x] Tick this box to confirm that a figure exemplifying the gating strategy is provided in the Supplementary Information.
